# Rosiglitazone Enhances Browning Adipocytes in Association with MAPK and PI3-K Pathways During the Differentiation of Telomerase-Transformed Mesenchymal Stromal Cells into Adipocytes

**DOI:** 10.3390/ijms20071618

**Published:** 2019-04-01

**Authors:** Abeer Maher Fayyad, Amir Ali Khan, Sallam Hasan Abdallah, Sara Sultan Alomran, Khalid Bajou, Muhammad Nasir Khan Khattak

**Affiliations:** 1 Department of Applied Biology, College of Sciences, University of Sharjah, Sharjah 27272, UAE; u15200250@sharjah.ac.ae (A.M.F.); u16101513@sharjah.ac.ae (S.S.A.); kbajou@sharjah.ac.ae (K.B.); mnasir@sharjah.ac.ae (M.N.K.K.); 2 Human Genetics & Stem cells research group, Research Institute of Sciences & Engineering, University of Sharjah, Sharjah 27272, UAE; sallam.abdallah@sharjah.ac.ae

**Keywords:** rosiglitazone, telomerase-transformed mesenchymal stromal cells (iMSC3), brown adipocytes, PPAR-γ, UCP-1, PI-3 kinase pathway, MAP kinase pathway

## Abstract

Obesity is a major risk for diabetes. Brown adipose tissue (BAT) mediates production of heat while white adipose tissue (WAT) function in the storage of fat. Roles of BAT in the treatment of obesity and related disorders warrants more investigation. Peroxisome proliferator activator receptor gamma (PPAR-γ) is the master regulator of both BAT and WAT adipogenesis and has roles in glucose and fatty acid metabolism. Adipose tissue is the major expression site for PPAR-γ. In this study, the effects of rosiglitazone on the brown adipogenesis and the association of MAPK and PI3K pathways was investigated during the in vitro adipogenic differentiation of telomerase transformed mesenchymal stromal cells (iMSCs). Our data indicate that 2 µM rosiglitazone enhanced adipogenesis by over-expression of PPAR-γ and C/EBP-α. More specifically, brown adipogenesis was enhanced by the upregulation of EBF2 and UCP-1 and evidenced by multilocular fatty droplets morphology of the differentiated adipocytes. We also found that rosiglitazone significantly activated MAPK and PI3K pathways at the maturation stage of differentiation. Overall, the results indicate that rosiglitazone induced overexpression of PPAR-γ that in turn enhanced adipogenesis, particularly browning adipogenesis. This study reports the browning effects of rosiglitazone during the differentiation of iMSCs into adipocytes in association with the activation of MAPK and PI3K signaling pathways.

## 1. Introduction

Obesity is one of the major risks for the development of complex and life-threatening metabolic disorders such as type 2 diabetes mellitus (T2DM) and cardiovascular diseases. It is a highly prevalent disorder characterized by excessive adipose tissues due to enhanced adipogenesis or increase in adipocyte size [1]. Therefore, it is crucial to study adipogenic differentiation from progenitor cells to mature adipocytes to identify molecular mechanisms and regulatory factors to effectively prevent and treat obesity and associated disorders [2].

Brown adipose tissue (BAT) and white adipose tissue (WAT) are both derived from mesenchymal stem/stromal cells [3]. BAT is characterized by multilocular droplets morphology, has a higher number of mitochondria and releases energy as heat by breaking down the triglycerides into free fatty acids through oxidative phosphorylation process. The BAT cells have a number of uncoupling protein-1 (UCP-1), which is part of the mitochondrial respiratory electron transport chain [4,5,6]. This protein is used as a marker for brown adipogenesis. UCP-1 increases respiration, oxygen consumption, and dissipates energy by thermogenesis [7]. Brown adipocytes have emerging roles in weight loss, regulation of glucose homeostasis and insulin sensitivity but some details at the cellular and molecular levels are poorly understood [8].

In contrast, WAT cells are larger in size and store energy as triglycerides. Excess white adipose tissue (WAT) is a main risk for causation of metabolic disorders such as obesity [8]. Insulin resistance developed by WAT is one of the pathogenic mechanisms causing obesity-linked type 2 diabetes mellitus [9]. Most of the pathogenic genes implicated in diabetes are associated with beta-cell function, whereas dysregulation of PPAR-γ is associated with insulin resistance [9,10].

Adipogenic differentiation involves modulation of gene expression by signaling pathways resulting in alteration of cellular type and morphology [11,12]. The differentiation occurs gradually starting with cell cycle arrest, determination, commitment, expression of markers and transcription factors, initial differentiation, terminal differentiation and maturation [13,14,15,16,17,18]. The differentiation of 3T3-L1, a pre-adipocyte cell line, into adipocytes is the most studied in vitro model of obesity [1]. Other in vitro models are mesodermal multipotent bone marrow-derived mesenchymal stromal cells (MSCs) that can undergo self-renewal as well as trilineage differentiation: osteoblasts, adipocytes, and chondrocytes [12]. The ease of availability and plasticity of bone marrow derived mesenchymal stromal cells make them attractive candidates to study adipogenic differentiation.

Nuclear receptors, peroxisome proliferator-activated receptors (PPARs), regulate different metabolic and catabolic processes during adipogenesis. It has three isoforms: PPARα, PPARβ/δ, and PPAR-γ. Among the three types, PPAR-γ is dominantly expressed in adipose tissue, being the master regulator of adipogenesis and having an essential role in glucose and fatty acid metabolism. Therefore, it is targeted by T2DM thiazolidinediones (glitazones) that bind to the nuclear PPAR-γ in diabetic treatment to increase insulin sensitivity [19].

Previous studies have reported that glitazones enhance adipogenesis and insulin signaling in 3T3-L1 cells by interaction with receptors of some secreted adipokines such as adiponectin [20]. These receptors activation indicate the activity of the adipokines in the presence of insulin and its responsibility for controlling glucose and lipid homeostasis [21]. Furthermore, it is also reported that rosiglitazone treatment instigates conversion of WAT into brown adipocytes in vitro [7]. However, no such effect of rosiglitazone has been reported during the differentiation of telomerase transformed mesenchymal stromal cells into adipocytes.

As reported in earlier studies, MAPK and PI3K signaling pathways have roles in adipogenesis [2,22,23,24]. A previous study shows that ERK1/2 pathway only has a role at the early adipogenesis [1]. However, the roles of MAPK/ERK and PI3K/AKT pathways at the maturation stage of brown adipogenesis during the differentiation of mesenchymal stromal cells (MSCs) into adipocytes has not been investigated. Therefore, molecular mechanisms linked to brown adipogenesis are of great interest in these stromal cells.

In this current study, we investigated the effects of rosiglitazone on the production of brown adipocytes during the in vitro adipogenic differentiation of telomerase-transformed MSCs. We evaluated the association of MAPK and PI3K pathways in the production of brown adipocytes during the differentiation.

## 2. Results

### 2.1. In Vitro Differentiation of Telomerase-Transformed MSCs into Adipocytes

**Figure 1:**
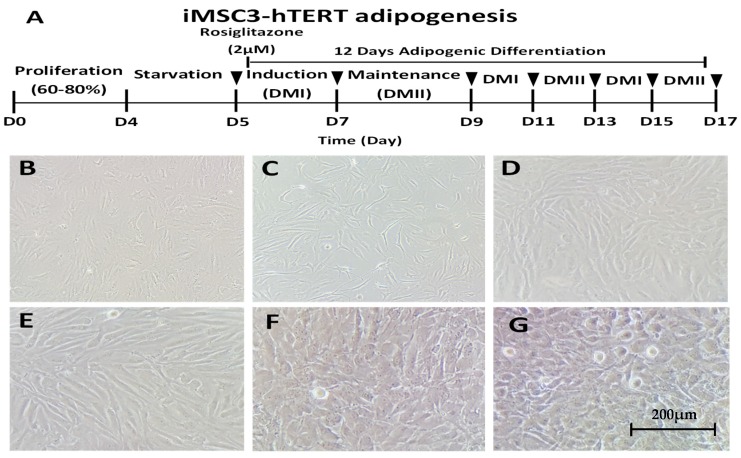
Differentiation of telomerase-transformed mesenchymal stromal cells into adipocytes: (**A**) adipogenic differentiation protocol of iMSC3-hTERT for three cycles (in total, 12 days) with and without rosiglitazone; (**B**) 50–60% confluent mesenchymal stromal cells; (**C**) cells after 24 h of serum starvation; differentiating adipocytes at the initial stages of induction (**D**) without and (**E**) with rosiglitazone; and mature adipocytes after 12 days of differentiation in control cells (**F**) and cells with rosiglitazone treatment (**G**).

To investigate the role of rosiglitazone in adipogenesis, we differentiated telomerase-transformed mesenchymal stromal cells into adipocytes without (−) and with (+) 2 μM of rosiglitazone following the protocol summarized in Figure 1A. The overall process and cellular morphology at the beginning and at the end are indicated in Figure 1. As shown in Figure 1B, iMSC3 cells have fibroblast-like morphology and grow by adhering to tissue culture flask. To start their differentiation, the cell cycle was synchronized by starving the cells in serum-free media (Figure 1C). At the initial induction stage, the cellular morphology started to change in both the control (Figure 1D) and treated (Figure 1E) cells. The morphological changes after induction indicate that adipocytes differentiation occurs. At the end of differentiation, mature adipocytes were formed with their lipid droplets vesicles formed in control (Figure 1F) and treatment (Figure 1G) conditions. This morphological change is a characteristic of mature adipocytes. We performed three differentiation cycles for a total 12-day interval to allow the complete differentiation of iMSC3 into adipocyte lineage, initially to pre-adipocyte and terminally into adipocytes.

### 2.2. Rosiglitazone Increases Fatty Acid Synthesis and Thus Lipid Content in Differentiated Adipocytes

**Figure 2:**
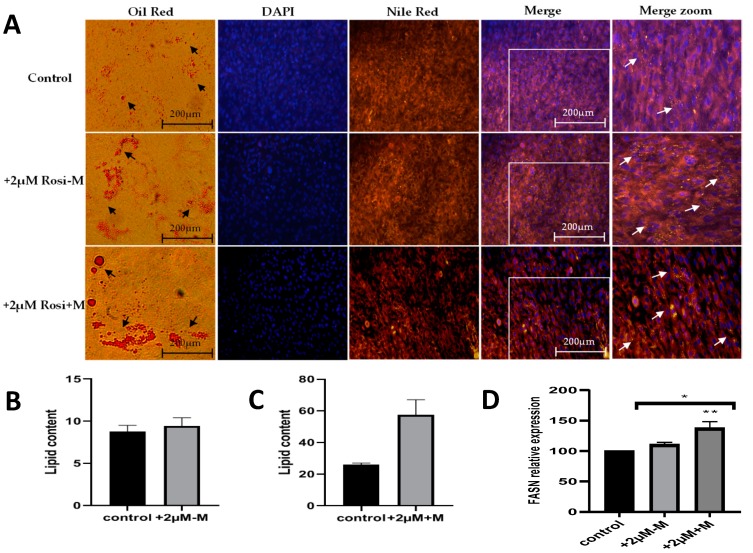
Effect of rosiglitazone on the fatty acid synthesis and lipid content in MSC-derived adipocytes. (**A**) Following differentiation, adipocytes from control, rosiglitazone in induction and rosiglitazone in both induction and maintenance were stained with Oil-O red, Nile red and DAPI stains to observe the increase in the density of lipid droplets accumulation in control, +2 µM-M, and +2 µM+M rosiglitazone, respectively. (**B**,**C**) Mature adipocytes lipid content was determined by eluting the Oil-O red stain and reading its absorbance at 500 nm. (**D**) Expression of fatty acid synthase gene was assessed, and data normalized to GAPDH gene. Data represent mean ± S.E.M. * *p* < 0.05, ** *p* < 0.01 versus control.

To study the formation of lipid vesicles under different experimental conditions, the oil red and fluorescent Nile red stains specific for cytoplasmic lipid droplets were used. Oil-O red, Nile red and DAPI stains (Figure 2A) show increased fatty droplets at the morphological level. DAPI dye specifically stains the nucleus, while nile red and oil red are specific for triglycerides and fats. As indicated by the arrows in Figure 2A, the staining intensity increased with 2 μM rosiglitazone treatment and increased further when rosiglitazone was present for 12 days (in the induction and maintenance media). Furthermore, the lipid content of the adipocyte cells was also determined. As indicated in Figure 2B,C, the lipid content increased under rosiglitazone treatment and was most increased when the rosiglitazone was present for 12 days (in both induction and maintenance), indicating that the rosiglitazone enhances the adipogenesis. For further confirmation of increased lipid content, the gene expression of fatty acid synthase gene (FASN) was studied. FASN is required for the synthesis of triglycerides and was significantly (** *p* < 0.01) increased after 48 h (rosiglitazone in induction and maintenance media) with rosiglitazone treatment compared to control (Figure 2D). No significant change was found after 96 h (rosiglitazone in induction only).

### 2.3. Rosiglitazone Enhances Adipogenic Differentiation by Targeting PPAR-γ and C/EBP-α in a Time-Dependent Manner

**Figure 3:**
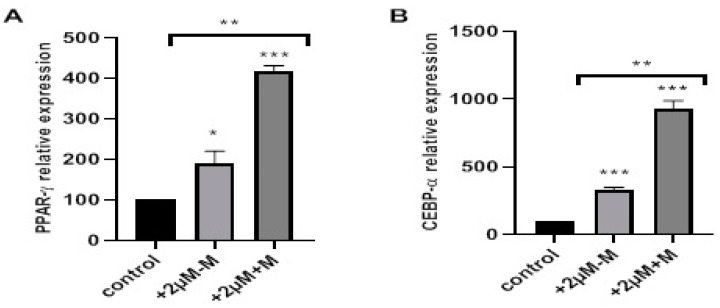
Effects of rosiglitazone on gene expression of PPAR-γ and C/EBP-α in mature adipocytes. Mesenchymal stromal cells were treated without (−) (control) or with (+) 2 µM rosiglitazone in both induction and maintenance media (+2 µM+M) or only in induction media (+2 µM-M) prior to RNA extraction. Extracted RNA was used for expression studies. The representative statistical data of (**A**) PPAR-γ and (**B**) C/EBP-α gene expression were normalized to GAPDH gene and are presented as mean ± S.E.M. * *p* < 0.05, ** *p* < 0.01, *** *p* < 0.001 versus control.

At the molecular level, we initially analyzed the genes PPAR-γ and C/EBP-α, which are the master regulators of adipogenesis, to confirm molecularly that the differentiation was enhanced in the presence of rosiglitazone, as shown in Figure 3. PPAR-γ was significantly overexpressed in adipocyte cells differentiated in the presence of 2 μM rosiglitazone in induction only (+2 μM-M) (* *p* < 0.05), and both in induction and maintenance (+2 μM+M) (*** *p* < 0.001). PPAR-γ expression increased by more than two folds in both treatment types. Consequently, C/EBP-α expression was also significantly enhanced in +2 μM-M and +2 μM+M conditions, as shown in Figure 3B (*** *p* < 0.001). Higher fold change was obtained for PPAR-γ and C/EBP-α when rosiglitazone was present in both induction and maintenance media, suggesting rosiglitazone mediated enhancement of adipogenesis is proportional to time exposure. PPAR-γ and C/EBP-α are regulators of adipogenesis and their upregulation clearly indicates that the adipogenesis was enhanced with the addition of rosiglitazone.

### 2.4. Rosiglitazone Enhances Browning Characteristics and Markers in Differentiated Adipocytes in a Time-Dependent Manner

**Figure 4:**
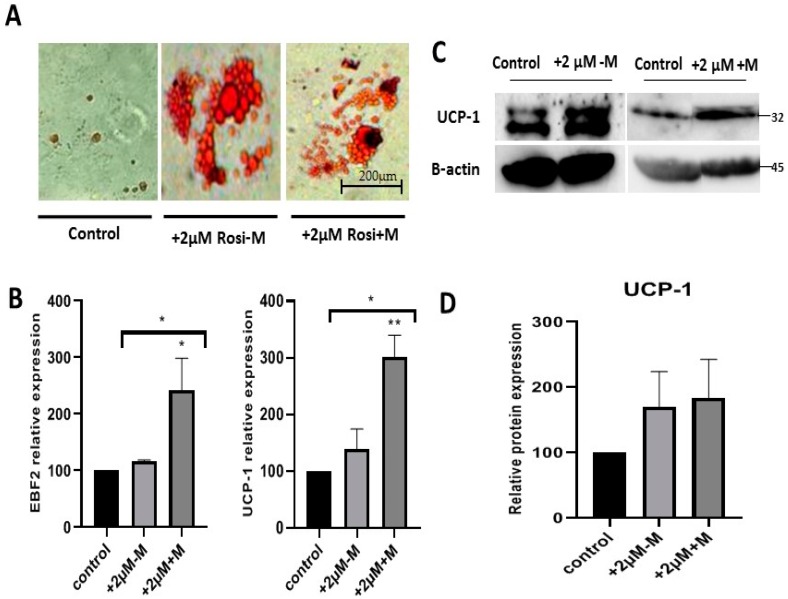
Rosiglitazone induces browning characteristics during adipocytes formation. (**A**) Oil-O red staining of adipocytes with (+) or without (−) rosiglitazone. (**B**) Gene expression profiles of EBF2 transcription factor and UCP-1 brown adipocyte marker in control and 2 µM rosiglitazone treatment in the induction (+2 µM-M) and rosiglitazone treatment within both induction and maintenance (+2 µM+M). (**C**) The representative Western blot of brown adipocyte marker, UCP-1, in the differentiated adipocytes without rosiglitazone and with rosiglitazone in the induction only and rosiglitazone in induction and maintenance: UCP-1 (~32 kDa), normalized to that of β-actin (~45 kDa). (**D**) Quantity of the UCP-1 protein level in each condition. The data are presented as mean ± S.E.M. * *p* < 0.05, ** *p* < 0.01 versus control.

To investigate the role of rosiglitazone in the production of brown adipocytes, Oil-O red stained adipocytes of different experimental groups were observed under bright field microscopy. The cells treated with rosiglitazone had multilocular lipid droplets morphology compared to the control (Figure 4A). To confirm the browning lineage, we studied the expression of the transcription factor EBF2 and the brown adipocyte marker UCP-1 (Figure 4B). Rosiglitazone induced browning of adipocytes during the differentiation, as EBF2 (* *p* < 0.05) and UCP-1 (** *p* < 0.01) genes expression significantly increased with rosiglitazone after 48 h exposure (+2 μM+M). To confirm the browning effect at the translational level, western blot was performed to detect the protein level of UCP-1 (Figure 4C). The UCP-1 was significantly increased, confirming overexpression at the transcriptional level (Figure 4D).

### 2.5. Rosiglitazone Enhances the Browning Effect by Activation of MAPK and PI3K Pathways

**Figure 5:**
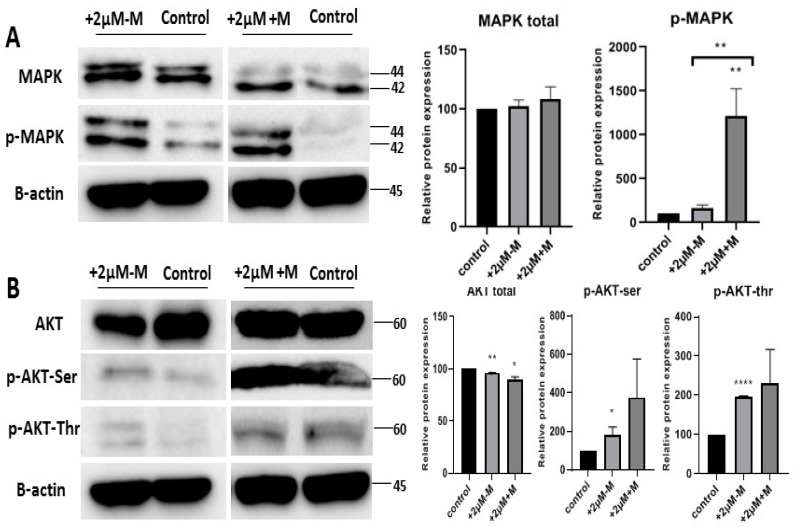
MAP kinase and PI3-kinase signaling pathways activated by rosiglitazone during the enhancing of brown lineage. Total and phosphorylated proteins of derived adipocytes from different experimental groups without (−) (control) or with (+) 2 µM rosiglitazone 48 hours (+2 µM+M) or 96 h (+2 µM-M): (**A**) MAPK (~42/44 kDa) and phospho-MAPK (~42/44 kDa); (**B**) total AKT (~60 kDa), phospho-AKT-serine (~60 kDa), and phospho-AKT-threonine (~60 kDa) normalized to that of β-actin (~45 kDa). Statistical data are presented as mean ± S.E.M. *p < 0.05, **p < 0.01, ***p < 0.001 versus control.

This study found that rosiglitazone enhanced brown lineages during the differentiation of telomerase-transformed MSCs into adipocytes (Figure 4). As MAPK and PI3-K signaling pathways are reported to be regulated during adipogenesis, we assumed that they might also play a role in brown adipogenesis under rosiglitazone treatment. Our data indicate upregulated phosphorylation of p-MAPK (Thr202/Tyr204) for MAPK pathway (Figure 5A); Serine-(Ser473) and threonine-(Thr308) for PI3-K pathway (Figure 5B) with rosiglitazone (2 μM) showing activation of MAPK and PI3-K signaling pathways (Figure 5). The phosphorylation of p-MAPK, Serine-(Ser473) and threonine-(Thr308) was more intense when rosiglitazone was present in both induction and maintenance media, indicating that rosiglitazone activated the MAPK and PI3-K pathways that in turn may activate genes that enhance the differentiation of brown adipocytes during the differentiation of iMSCs into adipocytes. 

## 3. Discussion

**Figure 6:**
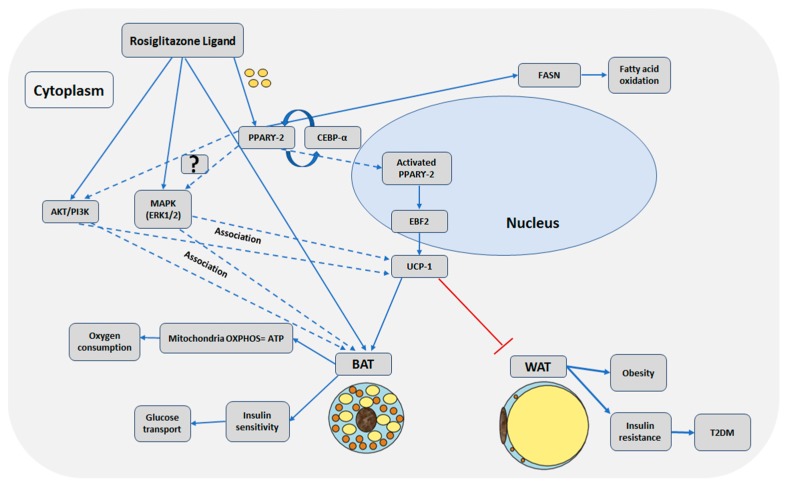
Schematic illustration of possible molecular mechanisms for Rosiglitazone enhanced browning of adipocytes. Solid line arrows indicate activation or induction, dotted line arrows indicate association that needs further confirmation of activation, and the red “T” arrow indicates inhibition.

In this study, we confirmed the in vitro browning of adipocytes with rosiglitazone treatment during the differentiation of telomerase transformed mesenchymal stromal cells into adipocytes. The activation of MAPK and PI3K pathways were associated with the browning adipogenesis. Figure 6 summarizes our results as well as findings from other studies.

Peroxisome proliferator-activated receptor (PPAR) regulates metabolic and catabolic processes and it has three isoforms: PPAR-α, PPAR-β/δ, and PPAR-γ [25]. Among them, PPAR-γ is dominantly expressed in adipose tissue, having an essential role in glucose and fatty acid metabolism. Therefore, it is targeted by T2DM drugs [19,25]. PPAR-γ has a Y-shaped pocket of one polar arm and two non-polar arms [25,26,27]. The polar arm is the major binding site for T2DM drugs, thiazolidinediones (TZD). Rosiglitazone, a thiazolidinedione, is an antidiabetic drug that enhances insulin sensitivity by activation of PPAR-γ [3,19,25,28]. This enhancement in sensitivity is without increasing the production of insulin by pancreas [19]. This enhancement increases adipogenic differentiation.

PPAR-γ along with C/EBP-α are key players in adipogenic differentiation that crosstalk and overlap to regulate the expression of target genes [17,26,29,30,31]. Their expression occurs at the late adipogenic stage and in mature adipocytes; these expressions are maintained in a positive feedback loop to preserve the phenotype of the mature adipocyte [29]. Most effects of TZD-treatment are through induction of adipogenesis, thus prompting lipogenic genes and consequently increasing the number of glucose transporters such as GLUT4 [20].

PPAR-γ and C/EBP-α are expressed in both BAT and WAT. They were overexpressed significantly with rosiglitazone treatment. In accordance with previous studies [31,32,33], our data indicate an enhanced effect on adipogenesis with PPAR-γ overexpression. Similarly, Ambele et al., who studied adipogenic differentiation of human adipose-derived stromal cells, showed that C/EBP-α and PPAR-γ genes were upregulated at the maturation stage of adipogenesis [32]. In another study on the differentiation of 3T3-L1 fibroblasts, Madsen et al. indicated that both PPAR-γ and C/EBP-α transcription factors co-operate to activate the adipogenic regulated genes [31]. In our study, the overexpression of PPAR-γ and C/EBP-α enhanced adipogenesis. The enhanced effect was confirmed morphologically and molecularly by increased lipid content and increased fatty acid synthesis evident by the upregulation of the fatty acid synthase gene (FASN).

In addition to enhancing general adipogenesis, rosiglitazone also enhances brown adipogenesis during the differentiation of iMSC3 into adipocytes. Browning of adipocytes was detected with distinct observed morphology and expression of UCP-1 and EBF2, markers of brown adipocytes. Brown adipocyte protein UCP-1 is expressed in mitochondria (10% of mitochondrial protein). It acts as an H^+^ proton leak in the mitochondrial inner membrane and increases mitochondrial uncoupled respiration upon activation [4]. Several studies also reported the interaction of EBF2, a transcription factor, with PPAR-γ enhancing the differentiation of the brown adipocytes [34,35,36]. Both EBF2 and UCP-1 were significantly upregulated with rosiglitazone treatment, indicating rosiglitazone enhanced browning adipogenesis during the differentiation of iMSCs into adipocytes. Our results confirm previous studies that report browning of adipocytes [8,37,38] and browning effects of rosiglitazone in adipose tissues of mouse [7,39]. According to the recent study by Sanchez-Gurmaches et al. [40], the lipogenesis gene FASN is correlated with UCP-1 and has a role in BAT [41]. However, in most of these studies, the differentiation of white pre-adipocytes cells into brown adipocytes is reported. In contrast, we differentiated the telomerase-transformed mesenchymal stromal cells into adipocytes.

Although the roles of PPAR-γ agonists such as rosiglitazone in the browning effect have been reported, the underlying molecular mechanisms such as signaling pathways need to be explored further. It is evident that, at the beginning of adipogenic differentiation, the adipogenesis specific transcription factors are activated. However, it seems that the browning related genes take longer to express, generally starting to be expressed on the seventh day and is being maintained until the end of the differentiation [7]. Therefore, we studied the pathways at the last stage of differentiation to allow time for browning genes to be expressed and then investigated the signaling pathways that are associated with the differentiation.

Our study found activation of MAPK and p-MAPK (Thr202/Tyr204) signaling pathways in the differentiation with the treatment of rosiglitazone. The differentiated cells were mostly brown adipocytes under rosiglitazone treatment. Hence, it suggests that MAPK might be involved in the enhancement of browning adipogenesis. Besides, the role of MAPK pathway, the enhanced expression of PI3-K/AKT and its phosphorylation sites at Serine 473 and Thr308 sites were also associated with enhanced brown adipogenesis.

Several previous studies indicated that MAPK pathway is downregulated in the early adipogenesis because it is associated with cellular expansion of cells which must be suppressed for adipogenesis [11,42]. However, in our study, at the late maturation stage of adipogenesis, the pathway was upregulated due to the rosiglitazone presence. Recently, Zhang et al. confirmed that the MAPK pathway enhances the browning of adipocytes during the differentiation of 3T3-L1 into adipocytes. However, this browning effect was mediated due to irisin [25]. Our study also indicated the possible involvement of MAPK in browning at the late stage of adipogenesis with rosiglitazone treatment. Another study on 3T3-L1 cells by Chen et al. confirms the role of MAPK in adipogenesis indicating that adipogenesis inhibition downregulates MAPK, PPAR-γ, C/EBP-α and decreases FASN and thus lipid accumulation [43].

A recent study on mice and human subcutaneous adipose tissue by Hong et al. [44] indicates the role of MAPK in the BAT function, showing that the activation of the MAPK (ERK1/2) pathway is crucial for the lipogenesis process, which provides free fatty acids as a fuel for brown adipocyte thermogenesis, whereas knock-out of ERK1/2 protein in vivo impairs lipolysis process. Additionally, upon β-adrenergic and norepinephrine stimulation, the ERK1/2 (MAPK) pathway is involved in the thermogenic process of BAT by enhancing cell proliferation and inhibiting apoptosis [40].

Than et al. [8], stated that the browning of white pre-adipose tissue is dependent on the suppression of the MAPK and activation of PI3-K pathway. In contrast, our study identified the activation of both pathways during the enhancement of brown adipogenesis. However, the study was performed on the differentiation of pre-adipose cells into adipocytes in mice and human in response to angiotensin. In contrast, we differentiated telomerase transformed MSC into adipocytes with rosiglitazone. Recently, in a study on obese ob/ob and wild type iBAT mice, Martins et al. showed decreased expression of FASN and altered insulin signaling by reduced phosphorylation of AKT at serine residues in obese mice [45]. Several studies report that PI3 kinase enhances adipogenesis and glucose uptake in the presence of insulin [8,11,21,46,47,48,49].

Furthermore, Chernogubova et al. reported that norepinephrine β_3_ adrenoreceptors increase glucose transport in brown adipocytes upon the activation of PI3-K pathway [50], indicating that glucose uptake and browning is dependent on AKT pathway. Standaert et al. confirmed that the combined action of insulin and rosiglitazone on 3T3-L1 adipocytes glucose uptake involves the insulin receptor and the PI3-K pathway activation [51]. Rat fetal brown adipocytes increase glucose uptake via GLUT4 and upregulation of PI3-K/AKT pathway when treated with rosiglitazone [52]. Another study supports previous studies while focusing on GLUT1 transporter in brown adipocytes and mTOR pathway [53].

In our work, rosiglitazone acted as a ligand that enhanced adipogenesis particularly brown adipogenesis through PPAR-γ in the presence of insulin in the media. Our study found the MAPK and PI3K are upregulated and activated by rosiglitazone that in turn might enhance adipogenesis, particularly brown adipogenesis, during the differentiation of telomerase transformed MSCs into adipocytes. Further studies should be carried out to differentiate the iMSCs into adipocytes in the presence of rosiglitazone and inhibitors of MAPK and PI3-K to confirm the effects of these pathways on the browning adipogenesis.

## 4. Materials and Methods

### 4.1. Cell Lines and Culture

Telomerase-transformed Immortalized Human Bone Marrow derived Mesenchymal Stromal Cells hTERT (iMSC3) (abm T0529, Canada) were used in this study. iMSC3 cells were maintained in complete Minimum Essential Medium Eagle (MEM) (Sigma-Aldrich M2279, St. Louis, MO, USA) containing 10% fetal bovine serum (FBS), 1% penicillin–streptomycin and 200 µM of L-glutamine. The cell culture was incubated at 37 °C in a humidified incubator with 5% CO_2_ supply.

### 4.2. Differentiation of MSCs into Adipocytes

The iMSC3 cells were differentiated into adipocytes using the protocol of Zebisch et al. [54] with slight modifications. iMSC3 cells were detached with 0.25% trypsin-EDTA and seeded into 6-well plates at a density of 5 × 10^4^ cells/well in the complete growth (MEM) media. The culture was maintained at 37 °C humidified incubator with 5% CO_2_. At 70–80% confluency, cells were subjected to serum starvation for 24 h to synchronize the cell cycle prior to differentiation [55,56]. The differentiation was carried out in three experimental groups: Group A, supplemented with 2 µM of Rosiglitazone in induction media only; Group B, supplemented with 2 µM rosiglitazone in induction and maintenance media throughout the differentiation for 12 days; and Group C, the control without rosiglitazone. Adipogenic differentiation was induced by induction medium I (DMI) containing 0.5 µM/mL 3-isobutyl-1-methylxanthine (IBMX) (Sigma-Aldrich, USA), 1 µM/mL Insulin (ITS) (Sigma-Aldrich, USA), 0.25 µM/mL Dexamethasone (Sigma-Aldrich, USA), 0.1 µM/mL Indomethacin (Sigma-Aldrich, USA) with or without 2 µM/mL rosiglitazone (Sigma-Aldrich, USA). After 48 h, the media was changed to differentiation maintenance media II (DMII) containing 1 µg/mL insulin (ITS) with or without 2 µM/mL rosiglitazone for 2 days [18,55,57,58,59]. The two-step adipogenic differentiation protocol was repeated for 3 cycles (12 days), and then the differentiated cells were studied to assess the role of rosiglitazone in adipogenic differentiation. The differentiated adipocytes were harvested for further analysis.

### 4.3. Nile Red and DAPI Staining

Nile red stain was prepared following the protocol of Greenspan et al. [60] with minor modifications. Nile red was prepared by dissolving 5 mg Nile red powder (Sigma-Aldrich N3013, USA) in 5 mL of acetone to obtain 1 mg/mL stock solution. Nile red stock solution was diluted in 1mM trizma-maleate (Sigma-Aldrich T3128, USA) and 3% *w/v* Polyvinylpyrrolidone (Sigma-Aldrich P2307, USA) to obtain 1:100 Nile red stain solution. Differentiation media was discarded, the adipocytes cells were washed with PBS and fixed with 4% paraformaldehyde for 1 h. Fixed cells were stained directly with 1:100 Nile red stain solution and DAPI stain (Life Technologies P36930, Eugene, OR, USA). Stained cells were imaged under the Olympus fluorescent microscope using Cellsens standard software.

### 4.4. Oil-O Red Staining

The Oil-O Red staining protocol was modified from Aguena et al. [61]. The cells were washed with PBS and then fixed with 4% paraformaldehyde for 1 h followed by incubation with 60% isopropanol for 15 min at room temperature. Isopropanol was discarded, and the cells were completely air dried. Cells were stained with Oil-O red solution (60% Oil- O red stock solution (Sigma-Aldrich 1391, USA), 40% distilled water) for 1 h at room temperature, and then washed with distilled water several times to remove any excess stain. Stained cells were imaged under the inverted microscope using Optika Vision lite software.

### 4.5. Lipid Content Determination

Lipid ratio was calculated from the different experimental groups as described in Aguena et al. [61]. Oil-O red stain was eluted from stained cells with 100% isopropanol at room temperature. Stain elution was put on a shaker for 15 min. Then, 100 μL of samples were quantified by ELISA 96-well plate reader at 500 nm absorbance (O.D.) to detect the readings for isopropanol eluted Oil-red stain for control and treated adipocytes. Isopropanol was used as the blank.

### 4.6. RNA Extraction and cDNA Synthesis

Total RNA was extracted from adipocytes using the Qiazol lysis reagent using miRNeasy extraction kit (Qiagen, Hilden, Germany) following the manufacturer protocol. The samples were quantified using Nanodrop 2000 spectrophotometer (Thermo Fisher Scientific, Waltham, MA, USA). cDNA was synthesized from total RNA using TruScript First Strand cDNA Synthesis Kit (Norgen Biotec, Thorold ON, Canada) following the manufacturer’s recommendations. The temperature profile was as follows: 25 °C for 5 min, 50 °C for 30–60 min, and 70 °C for 15 min.

### 4.7. Gene Expression Study by RT-PCR

Real time gene expression was carried out to assess the effects of rosiglitazone on adipogenic regulatory genes involved in brown and white adipogenic differentiation and insulin sensitivity. The qPCR primer sequences for target genes (PPAR-γ, C/EBP-α, FASN, EBF2, and UCP-1) and the house keeping gene GAPDH are shown in Table 1. Qiagen rotor gene thermal cycler (Qiagen, Germany) was used to perform real time gene expression using Go Taq qPCR Master Mix (Promega, Madison, WI, USA). The results were analyzed using Qiagen rotor gene qPCR software (Qiagen, Germany) and delta-delta Ct calculation method (Livak and Schmittgen, 2001) [62].

### 4.8. Protein Extraction and Immunoblotting

Harvested adipocytes cells were subjected to lysis buffer to obtain cell proteins. Triple adipocytes from three independent experiments were treated with detergent lysis buffer containing 50 mM Tris-HCl, 100 mM sodium chloride (NaCl), 0.25% sodium dodecyl sulfate (SDS), 0.1% sodium deoxycholate, 0.25% Triton X-100, 2 mM ethylenediaminetetraacetic acid (EDTA), phosphatase and protease inhibitor. Samples were centrifuged to remove the pelleted cellular debris. Protein concentrations were quantified by Bradford assay using Bio-Rad DC Protein Assay kit (Biorad 500-0116, Hercules, CA, USA) following manufacturer instructions.

Immunoblotting was performed to determine the differential regulation of MAP kinase and PI3 kinase pathways during the enhanced brown adipogenesis due to rosiglitazone. Prior to acrylamide gel electrophoresis, protein samples were normalized to 50 μg/μL and boiled for 5 min with B-mercaptoethanol for denaturation. Then, total protein samples were resolved on 12% SDS-PAGE gel. Proteins were transferred onto a nitrocellulose membrane (Bio-rad, Hercules, CA, USA) using a semi-dry blotting apparatus (Bio-rad, USA). The membranes were blocked at room temperature with 5% skimmed milk in TBS with 1% Tween-20, and probed overnight with the following primary antibodies: anti- β-actin (1:1000) (Cell signaling technologies 4970, USA), anti-Akt (1:1000) (Cell signaling technologies 9272, USA), anti-phospho-Akt (Thr308) (1:1000) (Cell signaling technologies 13038, USA), anti-phospho-Akt (Ser473) (1:1000) (Cell signaling technologies 9271, USA), anti-p44/42 MAPK (Erk 1/2) (1:1000) (Cell signaling technologies 4696, USA), anti-phospho- p44/42 MAPK (Erk 1/2) (1:1000) (Cell signaling technologies 4370, USA), and anti-UCP-1 (1:1000) (Thermofisher scientific PA1-24894, USA). After 3 washes with TBST, the blots were incubated with horseradish peroxidase (HRP) conjugated secondary antibody (1:1000) for 1 h. Enhanced chemiluminescent detection was performed using ECL solution (Bio-rad, USA) and the protein bands were (photographed using Geldoc system, complete name, and company name) quantified and analyzed on image Lab software (Bio-rad, USA).

### 4.9. Statistical Analysis

All experiments were performed in triplicates and results expressed as the means ± standard deviation or means ± standard error of the mean of the independent triplicate assays. Statistical significance was analyzed using GraphPad Prism 8 software by one-way ANOVA or two-tailed T-test considering significance at *p*-value <0.05.

## 5. Conclusions

Our study indicates that rosiglitazone enhanced expression of PPAR-γ that in turn enhanced adipogenesis, particularly browning effects. This enhancement might be mediated through the activation of both the MAPK and PI3-kinase pathways at the late stage of the differentiation with rosiglitazone treatment. These pathways are associated with maintaining the brown adipocyte phenotype. This suggests that there might be crosstalk between these two pathways at the maturation stage in adipogenic differentiation. 

## Figures and Tables

**Table 1:** qPCR primer sequences of target genes.

Gene	Forward Primer Sequence (5′-3′)	Reverse Primer Sequence (5′-3′)
**PPAR-γ**	5′-TTCTCCTATTGACCCAGAAAGC-3′	5′-CTCCACTTTGATTGCACTTTGG-3′
**C/EBP-α**	5′-TCGGTGGACAAGAACAG-3′	5′-GCAGGCGGTCATT-3′
**FASN**	5′AAGGACCTGTCTAGGTTTGATGC-3′	5′- TGGCTTCATAGGTGACTTCCA -3′
**EBF2**	5′-TAGGAAGAGGACCAACTCTGAAA-3′	5′-CGACATTAGCGTCCACCACTC-3′
**UCP-1**	5′-AGGTCCAAGGTGAATGCCC-3′	5′-TTACCACAGCGGTGATTGTTC-3′
**GAPDH**	5′-AGGGCTGCTTTTAACTCTGGT-3′	5′- CCCCACTTGATTTTGGAGGGA-3′

## Abbreviations

MSCs	Mesenchymal Stromal Cells
iMSC3	Immortalized Human Bone Marrow Mesenchymal Stromal Cells- hTERT
PPAR-γ	Peroxisome proliferator-activated receptor gamma 2
C/EBP-α	CCAAT/enhancer binding protein alpha
EBF2	Early B cell factor 2
UCP-1	Uncoupling protein 1
FASN	Fatty acid synthase
MAPK	Mitogen-activated protein kinases
PI3K/AKT	Phosphoinositide 3-kinase/Protein Kinase B

## References

[1] Kwak D.H., Lee J.H., Kim T., Ahn H.S., Cho W.K., Ha H., Hwang Y.H., Ma J.Y. (2012). Aristolochia Manshuriensis Kom Inhibits Adipocyte Differentiation by Regulation of ERK1/2 and Akt Pathway. PLoS ONE.

[2] Choi J., Kim J., Ali M., Jung H., Min B., Choi R., Kim G., Jung H. (2015). Anti-Adipogenic Effect of Epiberberine is Mediated by Regulation of the Raf/MEK1/2/ERK1/2 and AMPKα/Akt Pathways. Arch. Pharm. Res..

[3] Koppen A., Kalkhoven E. (2010). Brown vs. White Adipocytes: The PPARc Coregulator Story. Koppen, A.; Kalkhoven, E. Brown vs white adipocytes: The PPARγ coregulator story. FEBS Lett..

[4] Lu Y., Fujioka H., Joshi D., Li Q., Sangwung P., Hsieh P., Zhu J., Torio J., Sweet D., Wang L. (2018). Mitophagy is Required for Brown Adipose Tissue Mitochondrial Homeostasis during Cold Challenge. Sci. Rep..

[5] Cedikova M., Kripnerová M., Dvorakova J., Pitule P., Grundmanova M., Babuska V., Mullerova D., Kuncova J. (2016). Mitochondria in White, Brown, and Beige Adipocytes. Stem Cells Int..

[6] Shabalina I., Petrovic N., de Jong J.A., Kalinovich A., Cannon B., Nedergaard J. (2013). UCP1 in Brite/Beige Adipose Tissue Mitochondria is Functionally Thermogenic. Cell Rep..

[7] Merlin J., Sato M., Chia L.Y., Fahey R., Pakzad M., Nowell C.J., Summers R.J., Bengtsson T., Evans B.A., Hutchinson D.S. (2018). Rosiglitazone and a Β3-Adrenoceptor Agonist are both Required for Functional Browning of White Adipocytes in Culture. Front. Endocrinol..

[8] Than A., Xu S., Li R., Leow M., Sun L., Chen P. (2017). Angiotensin Type 2 Receptor Activation Promotes Browning of White Adipose Tissue and Brown Adipogenesis. Signal Transduct Target Ther..

[9] Ridderstråle M., Groop L. (2009). Genetic Dissection of Type 2 Diabetes. Mol. Cell. Endocrinol..

[10] Groop L., Pociot F. (2014). Genetics of Diabetes – are we Missing the Genes Or the Disease?. Mol. Cell. Endocrinol..

[11] Scioli M.G., Bielli A., Gentile P., Mazzaglia D., Cervelli V., Orlandi A. (2014). The Biomolecular Basis of Adipogenic Differentiation of Adipose-Derived Stem Cells. Int. J. Mol. Sci..

[12] Cook D., Genever P. (2013). Regulation of Mesenchymal Stem Cell Differentiation. Adv. Exp. Med. Biol..

[13] Janderová L., McNeil M., Murrell A.N., Mynatt R.L., Smith S.R. (2003). Human Mesenchymal Stem Cells as an in Vitro Model for Human Adipogenesis. Obes. Res..

[14] MacDougald O.A., Rosen E.D. (2006). Adipocyte Differentiation from the Inside Out. Nat. Rev. Mol. Cell Biol..

[15] Morrison R.F., Farmer S.R. (2000). Hormonal Signaling and Transcriptional Control of Adipocyte Differentiation. J. Nutr..

[16] Muruganandan S., Roman A., Sinal C. (2009). Adipocyte Differentiation of Bone Marrow-Derived Mesenchymal Stem Cells: Cross Talk with the Osteoblastogenic Program. Cell. Mol. Life Sci..

[17] Ntambi J.M., Young-Cheul K. (2000). Adipocyte Differentiation and Gene Expression. J. Nutr..

[18] Qian S.W., Li X., Zhang Y.Y., Huang H.Y., Liu Y., Sun X., Tang Q.Q. (2010). Characterization of Adipocyte Differentiation from Human Mesenchymal Stem Cells in Bone Marrow. BMC Dev. Biol..

[19] Sauer S. (2015). Ligands for the Nuclear Peroxisome Proliferator-Activated Receptor Gamma. Trends Pharmacol. Sci..

[20] Kudoh A., Satoh H., Hirai H., Watanabe T. (2011). Pioglitazone Upregulates Adiponectin Receptor 2 in 3T3-L1 Adipocytes. Life Sci..

[21] Bou M., Todorčević M., Rodríguez J., Capilla E., Gutiérrez J., Navarro I. (2014). Interplay of Adiponectin, TNFα and Insulin on Gene Expression, Glucose Uptake and PPARγ, AKT and TOR Pathways in Rainbow Trout Cultured Adipocytes. Gen. Comp. Endocrinol..

[22] Han J., Choi H.Y., Dayem A.A., Kim K., Yang G., Won J., Do S.H., Kim J., Jeong K., Cho S. (2017). Regulation of Adipogenesis through Differential Modulation of ROS and Kinase Signaling Pathways by 3,4′-Dihydroxyflavone Treatment. J. Cell. Biochem..

[23] Kim H., Sakamoto K. (2012). (−)-Epigallocatechin Gallate Suppresses Adipocyte Differentiation through the MEK/ERK and PI3K/Akt Pathways. Cell Biol. Int..

[24] Wijesekara N., Krishnamurthy M., Bhattacharjee A., Suhail A., Sweeney G., Wheeler M.B. (2010). Adiponectin-Induced ERK and Akt Phosphorylation Protects Against Pancreatic Beta Cell Apoptosis and Increases Insulin Gene Expression and Secretion. J. Biol. Chem..

[25] Da Silva F.M.C., dos Santos J.C., Campos J.L.O., Mafud A.C., Polikarpov I., Figueira A.C.M., Nascimento A.S. (2013). Structure-Based Identification of Novel PPAR Gamma Ligands. Bioorg. Med. Chem. Lett..

[26] Zoete V., Grosdidier A., Michielin O. (2007). Peroxisome Proliferator-Activated Receptor Structures: Ligand Specificity, Molecular Switch and Interactions with Regulators. Biochim. Biophys. Acta..

[27] Festuccia W.T., Blanchard P., Deshaies Y. (2011). Control of Brown Adipose Tissue Glucose and Lipid Metabolism by PPARγ. Front. Endocrinol..

[28] Choi J.H., Banks A.S., Estall J.L., Kajimura S., Bostrom P., Laznik D., Ruas J.L., Chalmers M.J., Kamenecka T.M., Bluher M. (2010). Obesity-Linked Phosphorylation of PPARγ by Cdk5 is a Direct Target of the Anti-Diabetic PPARγ Ligands. Nature.

[29] Siersbaek R., Nielsen R., Mandrup S. (2010). PPARγ in adipocyte differentiation and metabolism - Novel insights from genome-wide studies. FEBS Lett..

[30] Hansson B., Rippe C., Kotowska D., Wasserstrom S., Säll J., Göransson O., Swärd K., Stenkula K.G. (2017). Rosiglitazone Drives Cavin-2/SDPR Expression in Adipocytes in a CEBPα-Dependent Manner. PLoS ONE.

[31] Madsen M.S., Siersbaek R., Boergesen M., Nielsen R., Mandrup S. (2014). Peroxisome Proliferator-Activated Receptor and C/EBP Synergistically Activate Key Metabolic Adipocyte Genes by Assisted Loading. Mol. Cell. Biol..

[32] Ambele M.A., Dessels C., Durandt C., Pepper M.S. (2016). Genome-Wide Analysis of Gene Expression during Adipogenesis in Human Adipose-Derived Stromal Cells Reveals Novel Patterns of Gene Expression during Adipocyte Differentiation. Stem Cell Res..

[33] Loft A., Forss I., Siersbæk M.S., Schmidt S.F., Larsen A.B., Madsen J.G.S., Pisani D.F., Nielsen R., Aagaard M.M., Mathison A. (2015). Browning of Human Adipocytes Requires KLF11 and Reprogramming of PPARγ Superenhancers. Genes Dev..

[34] Rajakumari S., Wu J., Ishibashi J., Lim H., Giang A., Won K., Reed R., Seale P. (2013). EBF2 Determines and Maintains Brown Adipocyte Identity. Cell Metab..

[35] Shapira S.N., Lim H., Rajakumari S., Sakers A.P., Ishibashi J., Harms M.J., Won K., Seale P. (2017). EBF2 Transcriptionally Regulates Brown Adipogenesis Via the Histone Reader DPF3 and the BAF Chromatin Remodeling Complex. Genes Dev..

[36] Wang W., Kissig M., Rajakumari S., Huang L., Lim H.W., Won K.J., Seale P. (2014). Ebf2 is a Selective Marker of Brown and Beige Adipogenic Precursor Cells. Proc. Natl. Acad. Sci. USA.

[37] Sidossis L., Porter C., Saraf M., Børsheim E., Radhakrishnan R., Chao T., Ali A., Chondronikola M., Mlcak R., Finnerty C. (2015). Browning of Subcutaneous White Adipose Tissue in Humans After Severe Adrenergic Stress. Cell Metab..

[38] Chen Y.C., Yu Y.H. (2018). The Potential of Brown Adipogenesis and Browning in Porcine Bone Marrow-Derived Mesenchymal Stem Cells 1. J. Animal Sci..

[39] Li Y., Li X., Jiang T., Fan J., Zheng X., Shi X., Yu T., Chu G., Yang G. (2017). An Additive Effect of Promoting Thermogenic Gene Expression in Mice Adipose-Derived Stromal Vascular Cells by Combination of Rosiglitazone and CL316,243. Int. J. of Mol. Sci..

[40] Nedergaard J., Wang Y., Cannon B. (2019). Cell Proliferation and Apoptosis Inhibition: Essential Processes for Recruitment of the Full Thermogenic Capacity of Brown Adipose Tissue. Biochim. Biophys. Acta..

[41] Sanchez-Gurmaches J., Tang Y., Jespersen N.Z., Wallace M., Martinez Calejman C., Gujja S., Li H., Edwards Y.J.K., Wolfrum C., Metallo C.M. (2018). Brown Fat AKT2 is a Cold-Induced Kinase that Stimulates ChREBP-Mediated De Novo Lipogenesis to Optimize Fuel Storage and Thermogenesis. Cell Metab..

[42] Marquez M.P., Alencastro F., Madrigal A., Jimenez J.L., Blanco G., Gureghian A., Keagy L., Lee C., Liu R., Tan L. (2017). The Role of Cellular Proliferation in Adipogenic Differentiation of Human Adipose Tissue-Derived Mesenchymal Stem Cells. Stem Cells Dev..

[43] Chen C., Chen Y., Chen H., Chuang W., Lin A., Tsai C., Huang C., Lii C. (2016). Andrographolide Inhibits Adipogenesis of 3T3-L1 Cells by Suppressing C/EBPβ Expression and Activation. Toxicol. Appl. Pharmacol..

[44] Hong S., Song W., Zushin P.H., Liu B., Jedrychowski M.P., Mina A.I., Deng Z., Cabarkapa D., Hall J.A., Palmer C.J. (2018). Phosphorylation of Beta-3 Adrenergic Receptor at Serine 247 by ERK MAP Kinase Drives Lipolysis in Obese Adipocytes. Mol. Metab..

[45] Martins F.F., Bargut T.C.L., Aguila M.B., Mandarim-de-Lacerda C.A. (2017). Thermogenesis, Fatty Acid Synthesis with Oxidation, and Inflammation in the Brown Adipose Tissue of Ob/Ob (−/−) Mice. Ann. Anat..

[46] Lee H., Li H., Jeong J.H., Noh M., Ryu J. (2016). Kazinol B from Broussonetia Kazinoki Improves Insulin Sensitivity Via Akt and AMPK Activation in 3T3-L1 Adipocytes. Fitoterapia.

[47] Choi K.H., Lee H.A., Park M.H., Han J.S. (2017). Cyanidin-3-Rutinoside Increases Glucose Uptake by Activating the PI3K/Akt Pathway in 3T3-L1 Adipocytes. Environ. Toxicol. Pharmacol..

[48] Soundharrajan I., Kim D.H., Srisesharam S., Kuppusamy P., Choi K.C. (2018). Limonene Enhances Differentiation and 2-Deoxy-D-Glucose Uptake in 3T3-L1 Preadipocytes by Activating the Akt Signaling Pathway. Evid. Based Complement Alternat Med..

[49] Kim E.K., Kim S., Kim C.D., Ha J.M., Yun S.J., Chung S.W., Hong K.W., Bae S.S. (2010). Transcriptional Activation of Peroxisome Proliferator-Activated Receptor-Γ Requires Activation of both Protein Kinase A and Akt during Adipocyte Differentiation. Biochem. Biophys. Res. Commun..

[50] Chernogubova E., Cannon B., Bengtsson T. (2004). Norepinephrine Increases Glucose Transport in Brown Adipocytes Via Β3-Adrenoceptors through a cAMP, PKA, and PI3-Kinase-Dependent Pathway Stimulating Conventional and Novel PKCs. Endocrinology.

[51] Standaert M.L. (2002). Cbl, IRS-1, and IRS-2 Mediate Effects of Rosiglitazone on PI3K, PKC-, and Glucose Transport in 3T3/L1 Adipocytes. Endocrinology.

[52] Hernandez R., Teruel T., Lorenzo M. (2003). Rosiglitazone Produces Insulin Sensitisation by Increasing Expression of the Insulin Receptor and its Tyrosine Kinase Activity in Brown Adipocytes. Diabetologia.

[53] Olsen J.M., Sato M., Dallner O.S., Sandström A.L., Pisani D.F., Chambard J.C., Amri E.Z., Hutchinson D.S., Bengtsson T. (2014). Glucose Uptake in Brown Fat Cells is Dependent on mTOR Complex 2–promoted GLUT1 Translocation. J. Cell Biol..

[54] Zebisch K., Voigt V., Wabitsch M., Brandsch M. (2012). Protocol for Effective Differentiation of 3T3-L1 Cells to Adipocytes. Anal. Biochem..

[55] Lee M., Wu Y., Fried S.K. (2012). A Modified Protocol to Maximize Differentiation of Human Preadipocytes and Improve Metabolic Phenotypes. Obesity (Silver Spring, Md.).

[56] Cao Z. (2012). Growth Arrest Induction of 3T3-L1 Preadipocytes by Serum Starvation and their Differentiation by the Hormonal Adipogenic Cocktail. J. Cell Animal Biol..

[57] Abdallah B.M., Haack-Sørensen M., Burns J.S., Elsnab B., Jakob F., Hokland P., Kassem M. (2005). Maintenance of Differentiation Potential of Human Bone Marrow Mesenchymal Stem Cells Immortalized by Human Telomerase Reverse Transcriptase Gene Despite of Extensive Proliferation. Biochem. Biophys. Res. Commun..

[58] Contador D., Ezquer F., Espinosa M., Arango-Rodriguez M., Puebla C., Sobrevia L., Conget P. (2015). Dexamethasone and Rosiglitazone are Sufficient and Necessary for Producing Functional Adipocytes from Mesenchymal Stem Cells. Exp. Biol. Med. (Maywood).

[59] Skårn M., Noordhuis P., Wang M., Veuger M., Kresse S.H., Egeland E.V., Micci F., Namløs H.M., Håkelien A., Olafsrud S.M. (2014). Generation and Characterization of an Immortalized Human Mesenchymal Stromal Cell Line. Stem Cells Dev..

[60] Greenspan P., Mayer E.P., Fowler S.D. (1985). Nile Red: A Selective Fluorescent Stain for Intracellular Lipid Droplets. J. Cell Biol..

[61] Aguena M., Fanganiello R.D., Tissiani L.A., Ishiy F.A., Atique R., Alonso N., Passos-Bueno M.R. (2012). Optimization of Parameters for a More Efficient use of Adipose-Derived Stem Cells in Regenerative Medicine Therapies. Stem Cells Int..

[62] Livak K.J., Schmittgen T.D. (2001). Analysis of Relative Gene Expression Data using Real-Time Quantitative PCR and the 2−ΔΔCT Method. Methods.

